# Electroconvulsive Therapy (ECT) in Bipolar Disorder Patients with Ultra-Rapid Cycling and Unstable Mixed States

**DOI:** 10.3390/medicina57060624

**Published:** 2021-06-15

**Authors:** Sergey Mosolov, Christoph Born, Heinz Grunze

**Affiliations:** 1 Moscow Research Institute of Psychiatry, 107076 Moscow, Russia; 2 Russian Medical Academy of Continuous Professional Education, 125993 Moscow, Russia; 3 Psychiatrie Schwäbisch Hall, 74523 Schwäbisch Hall, Germany; c.born@klinikum-weissenhof.de (C.B.); heinz.grunze@icloud.com (H.G.); 4 Paracelsus Medical University, 90419 Nuremberg, Germany

**Keywords:** anticonvulsants, bipolar disorder, catatonia, ECT, lithium, mixed states, ultra-rapid cycling

## Abstract

*Background and Objectives:* Unstable mixed episodes or rapid switching between opposite affective poles within the scope of short cycles was first characterized in 1967 by S. Mentzos as complex polymorphous states with chaotic overlap of manic and depressive symptoms. Well-known examples include antidepressant-induced mania/hypomania and rapid/ultra-rapid/ultradian cycling, when clinicians observe an almost continuous mixed state with a constant change of preponderance of manic or depressive symptoms. Achieving stable remission in these cases is challenging with almost no data on evidence-based treatment. When mood stabilizers are ineffective, electroconvulsive therapy (ECT) has been suggested. Objectives: After reviewing the evidence from available literature, this article presents our own clinical experience of ECT efficacy and tolerability in patients with ultra-rapid cycling bipolar disorder (BD) and unstable mixed states. *Materials and Methods:* We conducted an open, one-year observational prospective study with a “mirror image” design, including 30 patients with rapid and ultra-rapid cycling BD on long-term mood stabilizer treatment (18 received lithium carbonate, 6 on valproate and 6 on carbamazepine) with limited effectiveness. A bilateral ECT course (5–10 sessions) was prescribed for regaining mood stability. *Results:* ECT was very effective in 12 patients (40%) with a history of ineffective mood stabilizer treatment who achieved and maintained remission; all of them received lithium except for 1 patient who received carbamazepine and 2 with valproate. Nine patients (30%) showed partial response (one on carbamazepine and two on valproate) and nine patients (30%) had no improvement at all (four on carbamazepine and two on valproate). For the whole sample, the duration of affective episodes was significantly reduced from 36.05 ± 4.32 weeks in the year prior to ECT to 21.74 ± 12.14 weeks in the year post-ECT (*p* < 0.001). Depressive episodes with mixed and/or catatonic features according to DSM-5 specifiers were associated with a better acute ECT response and/or long-term mood stabilizer treatment outcome after ECT. *Conclusions:* ECT could be considered as a useful option for getting mood instability under control in rapid and ultra-rapid cycling bipolar patients. Further randomized trials are needed to confirm these results.

## 1. Introduction

The term and dimensional concept of “mixed states” (MSs) was first proposed by Kraepelin [1] and Weygandt [2] in the late 19th century but has been effectively forgotten with the introduction of the bipolar–unipolar dichotomy in the Diagnostic and Statistical Manuals, 3rd and 4th edition (DSM-III [3]), (DSM-IV [4]). The revival of diagnosing and classifying MSs happened with the introduction of the mixed specifier in the Diagnostic and Statistical Manuals, 5th edition (DSM-5) [5], which drove the shift back toward a dimensional spectrum conceptualization. The separate category of a mixed episode in DSM-III and IV (meeting full diagnostic criteria for both a manic and depressive episode for at least 1 week) has been abandoned. The new criteria allow a diagnosis of mixed mania (MM) or mixed depression (MD) in bipolar disorder (BD) as well as a diagnosis of MD in unipolar depression. Apparently, the introduction of the mixed features specifier has increased the prevalence of MSs in clinical practice [6], urgently calling for clinical trials and the development of new recommendations for the treatment of MSs [7]. Mixed features are associated with worse outcomes and treatment resistance [8,9], higher risk of suicide [10,11], aggressiveness [12], affective lability [13], higher rates of recurrences [14,15], inversion to overt mania or hypomania [16], and increased cyclicity [17].

The incidence of classical rapid cycling (RC) (four or more episodes during a year) differs broadly in various BD populations—from 13% to 50% (with an average of 15% in non-selected populations) [18,19,20,21,22,23]. Case reports of ultra-rapid cycling (URC) with four or more episodes during a month, ultradian cycling (UDC) with four or more days of daily mood switches or even ultra-maximal cycling with hourly mood changes [24], which are almost impossible to differentiate from genuine MSs, occur sporadically in the literature, but the prevalence of such states is largely unknown [25,26,27,28]. The survey of 674 BD outpatients from the Stanley Foundation bipolar treatment outcome network revealed a rate of RC as high as 42%, of URC 26.8% and of UDC 19.7% [29,30].

When closely monitoring bipolar patients with RC, unstable mixed states (UMSs) are frequently observed in the process of switching or inversion from one affective pole to another [31,32]. Common phenomena are antidepressant-induced mania/hypomania and alternating (so-called “coupled”) phases or rapid/ultra-rapid/ultradian cycling, when clinicians observe almost continuous MSs with a constant change of predominance of manic or depressive symptoms [33,34,35].

Even in the early days of the nosological dichotomy, in contrast to the rather eclectic approach of Weygandt [2] and Kraepelin [1], Stransky proposed in 1911 to distinguish MSs not only by their mixture of manic and depressive symptoms but also by the order of clinical development (i.e., simultaneous—with dominant mania or depression, or successive—with a consecutive replacement of elements of opposite polarity within the same cycle) [36]. The most accurate distinction between stable and unstable mixed episodes (rapid switching of opposite affective phases within the scope of short cycles) was introduced by Mentzos [37]. He considered UMSs to be highly complex polymorphous conditions with a chaotic overlapping of manic and depressive symptoms that could not be captured due to their superposition.

Many authors regard affective instability as a predictor of illness progression and as an unfavorable outcome. Catatonic symptoms are more frequently observed in patients with MSs [38]. In line with this are the views of Janzarik from Germany [39,40] and Berner from Austria [41,42]. Both authors underline that all clinical forms of “dynamic emotional instability” such as UMSs predispose to the development of more complex psychotic states with perplexity, indecisiveness, perceptual disturbances, depersonalization, ideas of reference, and other sensory delusions. According to Berner, these psychopathological phenomena are holistic and specific to bipolar MSs and cannot be derived from a mere combination of depressive and manic symptomatology. Moreover, if the emotional instability is sustained, it leads to a “stable mixed state” (Vienna Research Criteria) characterized by the “persistent presence of a drive state, contradictory to the mood state and/or the emotional resonance” [42].

The International Classification of Diseases, 10th edition (ICD-10, [43]) has formally allowed the diagnosis of a mixed episode in the case of rapid alternation of manic, hypomanic, and depressive symptoms, but the different sets of symptoms must both be prominent for the greater part of the current episode and last for at least 2 weeks. States with subthreshold symptoms or of shorter duration were not considered for classification. In DSM-IV-TR, there was no diagnostic niche for such patients because they often do not meet the full criteria of a mixed episode or RC specifier, where episodes must be demarcated by 2 months of remission or a complete switch to an episode of opposite polarity [44]. With the release of the new DSM-5 mixed features specifier, the diagnostic strategy for these cases did not change significantly except for taking into account the subsyndromal states of the opposite polarity; because UMSs tend to be much shorter in duration than index episodes, they are not recognized for classification purposes [5]. It is no surprise, therefore, that UMSs are underdiagnosed and rarely treated adequately.

Achieving remission and maintaining stability in these cases is a challenging task. Management of unstable bipolar patients is a controversial issue with almost no evidence-based data. Common recommendations include avoiding antidepressants and using anticonvulsants (valproate/carbamazepine/lamotrigine) since lithium has been considered ineffective in these patients [7,45,46]. If mood stabilizers in general are ineffective, electroconvulsive therapy (ECT) has been recommended [47,48]. ECT is a primary choice for patients with refractory bipolar depression [49,50,51,52] and suicidal behavior [53,54,55]. It is also well known that ECT shows a rapid effect in patients with severe mania who are resistant to lithium and antipsychotic treatment, especially in cases of delirious mania or excited catatonia [56,57,58].

The evidence for using ECT in MSs is quite limited compared with that for its use in pure mania or depression. Curiously enough, the pioneers of ECT in clinical practice, Bini and Cerletti, applied it 80 years ago initially in a patient with acute MSs [59]. Most researches have rarely focused on ECT in MSs patients and have often not provided conclusive data on the predominant affect. Some studies demonstrated better efficacy of ECT in patients with syndromes close to DSM-5 “mixed depression”, another one close to “mixed mania” [60]. Most studies were retrospective analyses of case series [61,62,63,64]. We did not find any randomized controlled clinical trials or comparative prospective studies clearly distinguishing between the two categories of MD and MM, based on the new DSM-5 mixed specifier. Many authors highlighted the primary reduction of depressive symptoms and inferior ECT response in MSs, with a higher rate of residual hypomanic symptoms in remission compared to that for bipolar depression [65]. Other studies, however, reported a better or similar level of response compared to bipolar depression [60,66,67].

Several case reports mention ECT-induced mania/hypomania or MM while treating bipolar depression or MD in bipolar patients, with a switch incidence as high as 24.8% [68,69,70,71,72,73]. The data on the ECT response in RC or URC patients with affective instability are even more sparse [74,75]. According to clinical reports, the majority of patients on mood stabilizes have maintained remission for at least a 6-month period after an ECT course [76,77]. Almost nothing is known about the impact of acute ECT on the further course and long-term outcomes in BD. Follow-up observations after ECT in patients with MSs are sparse and limited to small case series that, however, demonstrated overall positive outcomes [67,78]. Nevertheless, there is little evidence to guide clinicians beyond these case reports and clinical experience.

Several studies investigated ECT as a potential intervention to maintain long-term euthymia in patients with BD, also called maintenance ECT (mECT), and showed that it was superior to pharmacotherapy alone [74,75,77,79,80,81].

The question of whether pharmacotherapy should be withdrawn during the ECT course is still up for debate [48,75,82,83]. Some authors consider ECT by itself to be a mood stabilizer with remission rates of 65.3% for pharmacotherapy-refractory depression and 88.0% for mania [53,68,84]. However, there is little evidence to support this notion.

Given the uncertainties about the best therapeutic approach to managing difficult-to-treat BD patients, we conducted an open prospective study with ECT add-on to a mood stabilizer using a “mirror” design. The objective of this study was to assess prospectively the short-term efficacy of ECT for different MSs (including UMSs), as well as the long-term (12 months) efficacy of previously ineffective mood stabilizer treatment after a single ECT course in bipolar patients with RC, and compare it to a retrospective period of the same duration (“mirror” design).

## 2. Materials and Methods

### 2.1. Participants

This is an open, observational, naturalistic, long-term, prospective, therapeutic study with a “mirror” design. The duration, intensity, and frequency of affective episodes were assessed for one year prior to and one year after the ECT course based on the patients’ reports and review of all available psychiatric records for every single patient, including an individual retrospective graphic chart as described by Post et al. [85]. ECT was applied to disrupt an unfavorable RC course of bipolar illness.

The design of the study was approved by the Local Ethics Committee of the Moscow Research Institute of Psychiatry (protocol number 18b, approved on 8 September 2014). All subjects gave their written informed consent to receive ECT and to participate in this study.

The study included patients diagnosed with type I BD (BD-I), according to DSM-IV, RC course (four and more affective episodes during the past year), 18–60 years old who continuously received one of three mood stabilizers (lithium carbonate, carbamazepine, or sodium valproate) during the last year, in adequate doses with therapeutic drug monitoring of plasma concentrations. For prophylactic purposes, the plasma level ranges of mood stabilizers were defined as follows: lithium 0.5–0.8 mmol/L, valproate 50–100 µg/mL, and carbamazepine 6–8 µg/mL. All patients were classified as non-responders to treatment in the year prior to ECT. After the ECT course, all patients continued to receive the same mood stabilizing treatment and were followed-up for the next 12 months. For assessing the efficacy of treatment, we applied either three (lithium, carbamazepine, valproate) or two (lithium and anticonvulsants) treatment groups to increase the statistical power of our sample.

The diagnosis of BD-I according to DSM-IV criteria was formally confirmed by the M.I.N.I. (Mini International Neuropsychiatric Interview—Russian version 5.0) [86]. All patients with other comorbid psychiatric diagnoses were excluded from the study.

According to the DSM-5 criteria of the “mixed features” specifier, MSs were grouped into two types—“mixed mania” (MM) and “mixed depression” (MD). Additionally, “major depressive episode” (MDE) and “catatonia specifier” (CS) were documented. Three catatonic symptoms out of twelve listed in the DSM-5 were sufficient for a CS diagnosis. A special clinical category of “unstable mixed states” (UMSs) was defined for those patients who had weekly or even daily frequent mood fluctuations (FMFs) and where it was impossible to determine the dominant affect for the past week.

### 2.2. ECT Procedure

Under general anesthesia with intravenous propofol (1.0–1.5 mg/kg) combined with the muscle relaxant succinylcholine (0.5–1.0 mg/kg) and atropine (0.5–1.0 mg), unilateral or bilateral ECT application was delivered using a pulse “ESTER” stimulator (TRIMA Ltd. Saratov, Russian Federation). Routine monitoring included pulse oximetry and electrocardiography. Stimulus setting was initially based on the “half-age” method, and >25 s was considered as a sufficient length for the seizures, measured as motor seizure duration. The stimulus intensity was chosen according to the titration at first treatment, the stimulus setting was then progressively raised (1.5–3 times) at the following sessions, the intensity was further elevated in case of inadequate seizure duration. Parameters included a pulse width of 1.0 ms, frequency ranging from 30 to 77 Hz, duration ranging from 2.0 to 5.0 s, and constant current interval of 550–900 mA with an electric charge of 130–160 mC. Patients were ventilated with 100% oxygen until resumption of spontaneous respiration. Valproate and carbamazepine were tapered before ECT and were paused during the ECT course. Lithium was not discontinued. The ECT course consisted of 5–10 sessions that were provided every other day excluding weekends. Unilateral ECT was provided initially in 20 patients using a right frontotemporal electrode placement. In eight of these patients with minimal or no improvement of the affective symptoms, a bilateral (bitemporal) treatment approach was used from the sixth to tenth ECT sessions. Bilateral ECT was used in 10 patients with severe affective symptoms, including catatonia and suicidal ideation, right from the beginning. The decisions regarding starting ECT and on electrode placement were made by the treating clinician after the patient did not respond to two adequate trials of medications with different pharmacological profiles, as an add-on to the mood stabilizer.

### 2.3. Measures

Response in acute MDE and MSs was defined as a rating of 2 “much improved” or 1 “very much improved” in the CGI-BP Improvement subscale (“Change from Preceding Phase, a—Mania, b—Depression”) [87] at the end of the ECT course.

The prophylactic efficacy of the ECT course was evaluated by comparing the two periods with stable and unchanged treatment (before and after the ECT course) applying a three-grade scale: (1) “effect”—≥75% reduction in number, duration, and severity of affective episodes; (2) “partial effect”—≥50% reduction in number, duration, and severity of affective episodes; and (3) “no effect”—<50% reduction in number, duration, and severity of affective episodes [88]. The ratings on this scale were checked against and verified by the second part of the CGI-BP (“Change from Preceding Phase, c—Overall Bipolar Illness”) [87], where a rating of 1—“very much improved” corresponded to a rating of 1—“Effect”, a rating of 2—“much improved” to a rating of 2—“Partial effect” and ratings of “Minimally improved” or “No change” or “Worth” to a rating of “No effect”.

An additional (secondary) measure of preventive mood stabilizing efficacy was the duration (in weeks) of affective symptomatology during one year prior to and one year after ECT. We could not calculate accurately other common outcomes, e.g., number of episodes or number of predominant manic or depressive episodes, because most patients had very short episodes with mixed symptoms.

### 2.4. Statistical Analysis

Data analysis was performed using SPSS statistical software version 21.0 (IBM Corp., Armonk, NY, USA). Descriptive analyses were used in terms of means and standard deviations for continuous variables and numbers and percentages for categorical ones. Sample characteristics and median levels of symptoms were compared using chi-square for categorial and Kruskal–Wallis for dimensional variables. Paired samples t-test was used to compare the duration of affective symptoms prior to and after ECT. The effect of three independent variables (four syndrome groups (major depressive episode (MDE), frequent mood fluctuations (FMFs), MD, MM), gender, type of therapy (lithium vs. anticonvulsants), and its interaction for the response (full and partial effect group and no effect group) was assessed with three-way ANOVA. The level of significance was set at *p* ≤ 0.05 (two-tailed) for all tests.

## 3. Results

The study recruited 30 bipolar patients, including 19 women (63.3%), aged 23 to 54 years (mean 41.0 ± 8.5 years) with a mean age at illness onset of 19.7 ± 4.7 years. Eighteen patients were on continuous long-term lithium carbonate treatment, six patients were on sodium valproate, and six patients were on carbamazepine. Although the number of participants in the anticonvulsant groups was three times smaller than in the lithium group, groups were at least largely comparable and did not significantly differ in main clinical and demographic indicators (Table 1). It is worth noting that there were no participants with an index MDE or MM in the valproate group, and four out of six patients suffered from FMFs and UMSs prior to the ECT course. In contrast, most patients on lithium had MDE or MM as the index episode.

Applying the DSM-5 criteria for the “mixed features” specifier at the start of the ECT course, six patients had “mixed depression” (MD), and four had “mixed mania” (MM). In ten patients, it was difficult to determine the predominant affect as they had frequent weekly or even daily FMFs or UMSs. Depression (MDE) as an index episode was observed in ten patients. Six patients met the DSM-5 criteria for CS (four of them had MD, one MDE, and one UMSs). Catatonic symptoms observed in our patient population were negativism, uncooperativeness, posturing and mannerism, rigidity, purposeless excitement with stereotypies, marked psychomotor retardation or stupor, and poverty of speech with echolalia. Most frequently, catatonic features were observed in patients with severe MD or UMSs.

The acute response rate to the ECT course according to the CGI-BP-I scale, as a function of different clinical and demographic characteristics, is presented in Table 2. The mental state of 18 patients (60%) improved much and very much (ratings 1 and 2) (15 patients were on lithium), and 9 patients (30%) achieved remission (rating 1) (6 patients were on lithium), with all affective symptoms fading rapidly. The remaining third of patients had negligible or no improvements. No variables were significantly associated with acute response, but we noted a tendency of worse ECT outcomes in MM and better responsiveness in patients with catatonic features (five of six patients responded). Almost all patients with depression and MD had a remarkable response to ECT: the mental condition of 9 out of 10 MDE patients and of all 6 patients with MD was much improved. However, in the MD patients, the improvement in depression was at the cost of a switch to MM or hypomania. We also observed ECT-induced hypomania in one patient diagnosed with MDE.

Separate comparative analysis of syndrome groups did not reveal any significant differences between the groups in demographic or illness characteristics, including the age of onset and prophylactic efficacy of mood stabilizer (Table 3). However, CS was significantly associated with severe MD, and the acute ECT response in bipolar patients was better in depression, MD, or UMSs, especially in the presence of catatonic symptoms (Table 3). Compared to other diagnostic groups, the acute ECT response was significantly better in MDE, but it was also satisfactory in MD and FMFs/UMSs.

The long-term efficacy of continuous mood stabilizer treatment after the ECT course, as a function of different clinical and demographic characteristics, is presented in Table 4. In total, during 12 months of treatment by the same, but previously ineffective mood stabilizer, the affective symptoms (“overall bipolar illness” by CGI scale) improved much or very much in 70% of RC bipolar patients with mood instability after a single ECT course: 12 patients (40%) maintained good remission, and 9 patients (30%) had a partial response with some residual affective symptoms.

The cumulative duration of affective symptoms in the year after the ECT was reduced significantly from 36.05 ± 4.32 to 21.74 ± 12.14 weeks (mean difference—14.30 ± 11.96) when compared to the same period prior to ECT (*p* < 0.001, t-paired test = 6.551). The least favorable result was observed in patients treated with carbamazepine, but there were no significant differences between the mood stabilizer groups: mean difference pre–post in the lithium group was 18.17 ± 10.96 weeks, in the carbamazepine group 5.63 ± 11.26 weeks, and in the valproate group 11.37 ± 11.79 weeks (chi-square = 5.069, *p* = 0.07).

There was no significant correlation between maintenance efficacy and available clinical and demographic variables, but, in general, lithium was slightly more effective than anticonvulsants (Table 4). The symptomatology of the index episode had no influence on mood stabilizer effectiveness in the long term. Nevertheless, patients with an MDE index episode seem to have more benefit from lithium prophylaxis. In addition, better outcomes were seen in patients with more severe index MDE or MD when criteria for CS were met. Conversely, if a patient had MM as an index episode, the long-term results were worse.

**Figure 1:**
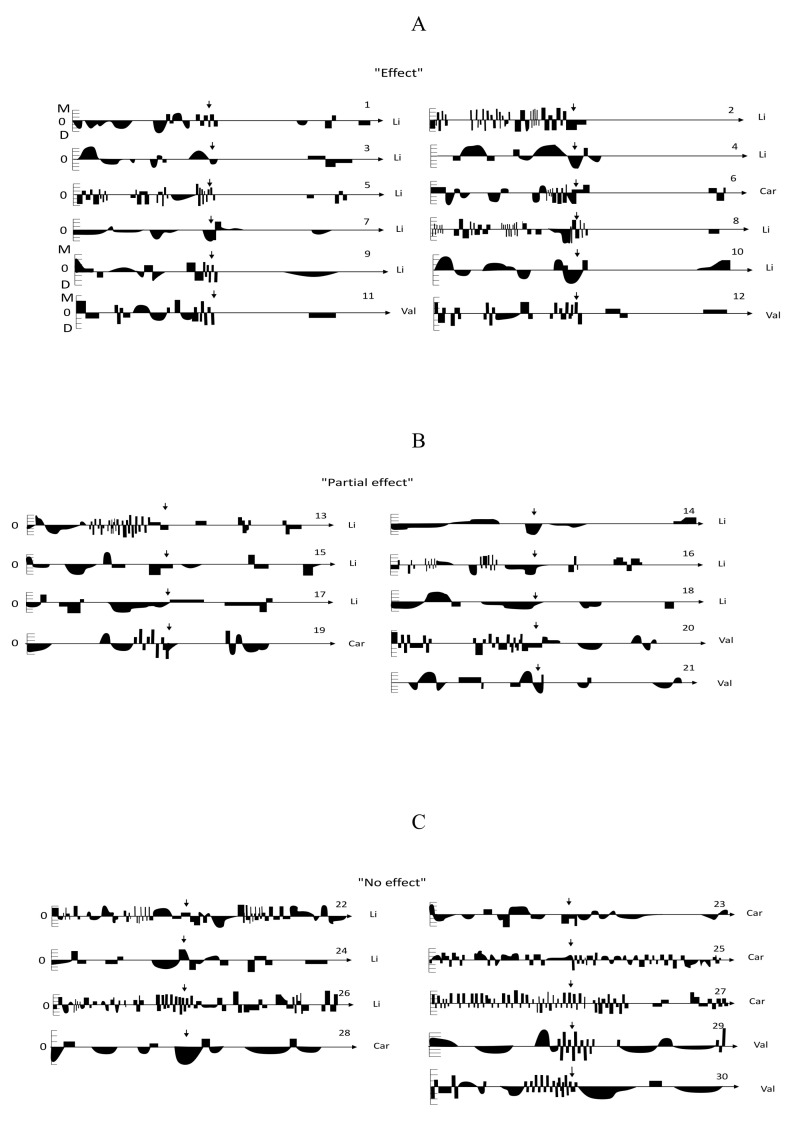
Individual graphic life charts of affective symptoms in the one year prior to (retrospective part) and the one year after (prospective part) the ECT course, grouped by the degree of response to the long-term mood stabilizing therapy. Footnote: Car—carbamazepine, ECT—electroconvulsive therapy, Li—lithium, Val—valproate. Axis X—severity: above 0—mania (mild, moderate, severe), below 0—depression (mild, moderate, severe). Axis Y—the duration of affective symptoms—12 months before and 12 months after the ECT, whose beginning is marked with an arrow. (**A**) Patients #1–12—very good response with the achievement of remission (“Effect” or very much improved as measured with the CGI-BP). (**B**) Patients #13–21—good response (“Partial effect” or much improved in the CGI-BP). (**C**) Patients #22–30—no response to long-term mood stabilizing therapy (“No effect”).

Patient-by-patient results are visualized in Figure 1 depicting two-year individual life charts. As shown, ECT was very effective in 12 patients, who were able to maintain euthymia despite having a history of previously ineffective mood stabilizer treatment, 9 of them were on lithium, 2 on valproate, and 1 on carbamazepine. At least six months of full remission with no residual symptoms was maintained in 11 patients; 6 patients had symptoms of minor depression in the second half of the year, and 4 patients showed brief hypomanias or MSs (two of them were on anticonvulsants) (Figure 1A). We observed a partial mood stabilizing effect after a single ECT course with less frequent residual affective symptoms including MSs in nine patients during one year follow-up, and all participants no longer had a RC/URC illness course (six patients were on lithium, two on valproate, one on carbamazepine) (Figure 1B). Figure 1C shows the life charts of nine patients who had no improvement at all (four on carbamazepine, three on lithium, and two on valproate), five of them continued to suffer from an unfavorable URC course.

We observed some features of switching (phase inversion) to MM or hypomania during ECT treatment in nine patients, and in all cases, the emergence of manic symptoms was very short (several days after ECT exposure, except for a longer hypomanic aftermath in patient #17): Five patients were on lithium, two on valproate, and two on carbamazepine. Six of them had an index episode of MD, and hypomania was only revealed after the reduction of depressive symptoms. Neither lithium nor valproate were able to prevent full phase inversion.

We tried to further explore potential factors influencing the response to long-term mood stabilizing therapy after the ECT course, combining the “full” and “partial” prophylactic effect group and comparing it to the “no effect” group. Three-way ANOVA revealed a significant influence of the index episode and the prognostic importance of distinguishing MDE from different MSs, including UMSs (Table 5). We did not find any significant impact on long-term response by gender or mood stabilizing agent (lithium vs. anticonvulsants, although there was a strong trend for a better general efficacy of lithium treatment) or their associations (interactions) with each other or with a syndrome. When we used one-way ANOVA the effect of the therapeutic agent just missed significance (F = 4.073, *p* = 0.053).

The safety and tolerability of the ECT and concomitant short-term anesthesia were good in all our patients. We observed reversible memory impairment in 12 patients (40%), but only three of them had cognitive symptoms (retrograde amnesia) lasting for more than a week. Additionally, two patients complained about severe headache, and one had prolonged apnea after anesthesia.

## 4. Discussion

The main result of this study is that a single ECT course was able to interrupt URC and mood instability in 40% of BD patients and, what is even more important, modify or even restore the prophylactic efficacy of a previously ineffective mood stabilizer. This evidence is especially important for lithium treatment, which is often considered a less effective agent than anticonvulsants in RC [45,89]. After a single ECT course, lithium treatment was again very effective in half of patients whose course was previously resistant to it, and another third of patients showed a partial benefit. The capability of ECT to improve the efficacy of anticonvulsant mood stabilizers was less evident, acute affective symptoms improved in only one patient on carbamazepine and two patients on valproate very much, and another one on carbamazepine and two patients on valproate, respectively, had a partial prophylactic benefit after a single ECT course. It appears that a period of stable euthymia, free of affective symptoms, is needed for lithium to be an effective treatment. The mechanism how ECT restores lithium effectiveness is still speculative. Possible explanations could be a modulation of the cell membrane ionotropic channel activity, neurotransmitter turnover, increase in brain-derived neurotrophic factor (BDNF) production, or a change in blood–brain barrier permeability [90,91,92]. An important finding for clinical practice is that a switch from lithium to anticonvulsant appears not always necessary in treatment-resistant RC. In several of our patients, ECT was sufficient to stop continuous cycling. We could not locate any literature reporting on a change of long-term lithium efficacy after a single, three-week ECT course ending continuous mood fluctuations.

Another relevant finding of this study is that the effect of acute ECT differed in various mixed states. Altogether, 13 (65%) of the 20 patients responded to ECT, which is in line with the response rates from other studies [47,60,93,94] and those reported in reviews [95]. The most pronounced response was in patients with MD (100% response rate) and unstable mixed states with FMFs (60% response rate). The most resistant to ECT were those with MM, only one out of four patients had a rating of “much improvement” in the CGI-BP. These data differ from the results of Ciapparelli’s study [60] but are by large in accordance with of the results of Medda et al. [93], who reported less response and higher final Young Mania Rating Scale (YMRS) scores in the MSs group. Although the response rate to ECT in MM was still high (56%), Ciapparelli et al. [60] also described a more pronounced initial reduction of depressive symptoms. Three of these patients in our study cohort were on lithium and one on carbamazepine, which is consistent with observations of other authors reporting poorer response to lithium in MM [96,97]. Our study also confirmed the high efficacy of ECT in bipolar depression: 9 out of 10 patients with depressive episodes had CGI-BP ratings of much or very much improvement, which is in accordance with several previous findings [98,99]. Moreover, severe depression with catatonic features showed a better response to ECT: five out of six patients were responders, with four of them having mixed features. In our study, catatonic features occurred much more often in patients with an index episode of MD. These data are in full accordance with several other recent studies [47,94]. However, in contrast to the study by Medda et al. [66], we did not find any significant differences between syndrome groups for gender, patient age, or age at BD onset.

It is worth mentioning that our findings support the phenomenological observations of Berner, who considered that dynamic emotional instability and UMSs predispose patients to developing more complex psychotic states with catatonic signs [42]. Because of the absence of diagnostic criteria and their polymorphous as well as volatile clinical presentation, UMSs are often misdiagnosed and are rarely treated adequately, also in respect to offering ECT. Following the previous generation of our European colleagues, we believe that along with new MD and MM categories, it is also important to distinguish UMSs, which not only have a different etiology (syndromokinesis) and phenomenology but also have a different prognosis with frequent rapid progression to more severe psychotic conditions and a different response to therapy. This diagnostic category represents a subset of especially difficult-to-treat bipolar patients. As our observations demonstrated, ECT was able to cease FMFs with UMSs and modify the response to mood stabilizer. Therefore, in order to achieve a rapid response in the most severe cases with an unfavorable BD course, we suggest that ECT should not only be used as a last resort in fully medication-resistant patients but should also be considered as a first- or second-line choice already in the early stages of FMF development.

Interestingly, and contrary to the data from earlier studies [47,100,101], we observed switching to hypomania/mania during the ECT course in seven patients with an index episode of depression, but six of them already had marked mixed features according to the DSM-5 specifier MD. After initial alleviation of the depressive symptoms in MD, the ECT brought manic symptoms to the surface. Therefore, the distinction between pure bipolar depression and MD is important for predicting an over-spilling ECT effect with the development of full-blown mania. Similarly, early recognition of mixed features in MDE may caution against excessive use of antidepressants, which is often clinical practice in such cases [102], even though the rational for their use should be well balanced in BD since they may provoke faster cyclicity and RC [103]. Of note, however, the duration of manic symptoms was rather short: in all cases except for one hypomanic patient, it lasted only for several days after ECT exposure, which is very similar to previous observations by Andrade et al. [104]. It is difficult to say whether switching was causally determined by ECT or just spontaneous residual cycling due to the natural history of BD. Moreover, caution should be exercised when differentiating between a treatment-induced manic or hypomanic episode and psychomotor agitation as a frequent transient side effect of ECT. Some reports exist that lithium can prevent ECT-induced mania [71], but in our study, lithium was ineffective in four patients (three of whom had MD prior to the ECT). Unlike anticonvulsants, we did not withdraw lithium prior to ECT sessions, and, in contrast to some reports that report an increase in side effects with concurrent use of lithium during ECT [105,106], our participants had no additional tolerability problems.

### Limitations

The study has several limitations. First of all, it is limited by scope, statistical power is low, and the sample size is insufficient for strong evidence or to use it in any kind of multifactorial predictive models. It should rather be considered as a case series from clinical practice, where the dynamics of clinical manifestations of each patient are important and worth individual discussions and reporting. In fact, most of the reviewed studies on the topic are naturalistic, observational, and consisted of case series. Secondly, there were methodological shortcomings such as the lack of a control group, no randomization, prospective observation only for a one-year follow-up period after ECT, and the non-blinded evaluations. However, the likelihood that a placebo effect or spontaneous remissions have significant influence on the results is quite low, given that all patients had a well-documented previous clinical history of severe affective symptoms and a long-standing continuous course, which was resistant to adequate mood stabilizing treatment, making these patients also not eligible for randomized controlled trials. Despite all the efforts to assess the severity of affective symptoms in the year preceding ECT (patient and relative interview, medical records, etc.) subjective interpretations cannot be excluded, and comparison of prospective and retrospective periods cannot be considered as equally reliable. In addition, we did not explore in great detail many clinical variables such as comorbidities, baseline manic symptom severity, duration of current episodes prior to ECT, etc., which might have an impact on the ECT response in MSs [93]. During the ECT course, we did not withdraw any other psychopharmacological agents except for anticonvulsants; therefore, any influence of the various medications on the response to the acute ECT cannot be fully excluded [83], and a control group of ECT alone should be added in further studies. Additionally, the interruption of long-term anticonvulsant therapy could have affected prophylactic efficacy. However, due to the reasonable follow-up observation period of one year and evident pharmacotherapy resistance prior to the ECT, the likelihood of the differences in pharmacological treatment impacting on the outcome is low. Finally, treatment groups were not comparable by size, as most of the participants were on lithium therapy, and the anticonvulsant groups were very small.

One more limitation, specific to Russia, is the difficulty associated with bipolar patients’ screening and problems with recruitment of participants in clinical practice. The diagnosis of BD in Russia is at least hundred times less frequent than in Western Europe; this is especially true for BD-II [107], where only one recently validated screening tool is available for the detection of hypomania in patients with recurrent depression [108,109]. This may explain the lack especially of BD-II patients in our sample, although RC is much more common in BD-II [23].

## 5. Conclusions

ECT can be considered as an effective and safe method for ceasing a URC course and mood instability including UMSs in BD-I patients resistant to mood stabilizers. Moreover, a single ECT course could restore the preventive mood stabilizing efficacy of long-term lithium treatment in half of those previously non-responsive to treatment, making a switch to alternative mood stabilizing treatments, e.g., anticonvulsants, unnecessary.

It also makes sense to delineate MM and MD according to the DSM-5 mixed features specifier from phenomenologically distinct UMSs that should be considered as a more complex transitional phenomena with a higher risk of developing severe psychotic states, including catatonic symptoms. Unlike psychopharmacotherapy, ECT is highly effective in this condition and can cut through continuous mood fluctuations.

Clearly, further research is needed, including randomized clinical trials, to substantiate the evidence of acute ECT efficacy in URC and UMSs patients, as well as its role in restoring long-term efficacy of lithium and other mood stabilizer treatments.

## Figures and Tables

**Table 1:** Demographic and clinical characteristics of the medication groups (lithium, carbamazepine, and valproate).

Parameter	Total (*n* = 30) (%)	Li (*n* = 18)	Car (*n* = 6)	Val (*n* = 6)	Chi-Square	*p*
Gender/Female, *n* (%)	19 (63.3)	11 (57.9)	4 (21.0)	4 (21.0)	0.096	0.953
Age, mean (±SD)	41.00 (8.45)	39.56 (8.99)	46.00 (5.59)	40.33 (8.36)	2.296	0.317
Age of onset, mean (±SD)	19.73 (4.69)	20.28 (4.84)	17.50 (4.23)	20.33 (4.76)	1.698	0.428
Duration of affective symptoms during the year prior to ECT (weeks), mean (±SD)	36.05 (4.32)	36.56 (3.69)	34.56 (4.36)	36.00 (6.26)	0.928	0.629
MM, *n* (%)	4 (13.3)	3 (75.0)	1 (25.0)	0 (0)	1. 154	0.562
MDE, *n* (%)	10 (33.3)	8 (80.0)	2 (20.0)	0 (0)	4.000	0.135
FMFs/UMSs, *n* (%)	10 (33.3)	4 (40.0)	2 (20.0)	4 (40.0)	4.000	0.135
MD, *n* (%)	6 (20)	3 (50.0)	1 (16.7)	2 (33.3)	0.833	0.659

Footnote: Car—carbamazepine, MDE—major depressive episode, FMFs—frequent mood fluctuations, Li—lithium, MD—mixed depression, MM—mixed mania, SD—standard deviation, UMSs—unstable mood states, Val—valproate.

**Table 2:** Acute ECT response measured with the CGI-BP-I scale in relation to demographic variables (female gender, age, age at onset), treatment, and illness characteristics (index episode, presence of catatonic specifier).

Parameter	Total (*n* = 30)	Very Much Improved (1) (*n* = 9)	Much improved (2) (*n* = 9)	No Improvement (*n* = 12)	Chi-Square	*p*
Gender/Female, *n* (%)	19 (100.0)	2 (15.8)	10 (52.6)	6 (31.6)	1.805	0.406
Age, mean (±SD)	41.00 (8.45)	41.43 (6.45)	39.67 (8.91)	43.13 (9.57)	0.950	0.622
Age of onset, mean (±SD)	19.73 (4.69)	21.00 (3.83)	20.07 (5.65)	18.00 (3.07)	1.501	0.472
Li vs. Car + Val, *n* (%)	18 (100.0)	6 (33.3)	9 (50.0)	3 (16.7)	3.616	0.164
MM, *n* (%)	4 (100.0)	0 (0.0)	1 (25.0)	3 (75.0)	5.697	0.058
MDE, *n* (%)	10 (100.0)	3 (30.0)	6 (60.0)	1 (10.0)	2.148	0.342
FMFs/UMSs, *n* (%)	10 (100.0)	3 (30.0)	3 (30.0)	4 (60.0)	2.486	0.289
MD, *n* (%)	6 (100.0)	1 (16.7)	5 (83.3)	0 (0.0)	3.810	0.149
CS, *n* (%)	6 (100.0)	1 (16.7)	4 (66.7)	1 (16.7)	0.841	0.657

Footnote: Car—carbamazepine, CGI-BP-I—Clinical Global Impression-Bipolar-Improvement, CS—catatonia specifier, ECT—electroconvulsive therapy, MDE—major depressive episode, FMFs—frequent mood fluctuations, Li—lithium, MD—mixed depression, MM—mixed mania, SD—standard deviation, UMSs—unstable mood states, Val—valproate.

**Table 3:** Demographic and illness characteristics, acute ECT response, and maintenance of efficacy in different index episode groups (MM, MDE, FMFs/UMSs, and MD).

Parameter	MM (N = 4) *n* (%)	MDE (N = 10) *n* (%)	FMFs/UMSs (N = 10) *n* (%)	MD (N = 6) *n* (%)	Chi-Square	*p*
Gender/Female, *n* (%)	3 (16.7)	5 (27.8)	7 (38.9)	3 (16.7)	1.458	0.692
Age, mean (±SD)	38.00 (12.94)	41.40 (7.59)	49.90 (7.34)	42.50 (10.11)	2.547	0.467
Age of onset, mean (±SD)	17.00 (3.56)	20.20 (5.39)	19.10 (3.90)	21.83 (5.27)	0.820	0.845
Li (vs. Car + Val), *n* (%)	3 (16.7)	8 (44.4)	4 (22.2)	3 (16.7)	3.958	0.266
CS, *n* (%)	0(0.0)	1 (16.7)	1 (16.7)	4 (66.7)	10.417	0.015 *
Acute ECT response by CGI-BP-I (1 + 2 vs. no improvement), *n* (%)	1 (4.5)	9 (40.9)	6 (27.3)	6 (27.3)	9.290	0.026 *
Long-term mood stabilizing effect (Effect + Partial effect vs. No effect), *n* (%)	1 (4.8)	8 (38.1)	6 (28.6)	6 (28.6)	7.381	0.061

Footnote: Car—carbamazepine, CGI-BP-I—Clinical Global Impression-Bipolar-Improvement, CS—catatonia specifier, ECT—electroconvulsive therapy, MDE—major depressive episode, FMFs—frequent mood fluctuations, Li—lithium, MD—mixed depression, MM—mixed mania, SD—standard deviation, UMSs—unstable mood states, Val—valproate. * *p* < 0.05.

**Table 4:** Demographic and clinical characteristics of groups by the level of response to long-term mood stabilizer treatment.

Parameter	Total (*n* = 30)	Effect (*n* = 12)	Partial Effect (*n* = 9)	No Effect (*n* = 9)	Chi-Square	*p*
Gender/Female, *n* (%)	19 (100.0)	7 (36.8)	6 (31.6)	6 (31.6)	0.215	0.898
Age, mean (±SD)	41.00 (8.45)	38.83 (8.70)	41.33 (7.63)	43.56 (9.04)	1.625	0.444
Age of onset, mean (±SD)	19.73 (4.69)	20.67 (3.87)	20.78 (6.30)	17.44 (3.32)	2.787	0.248
Duration of affective symptoms during the year prior to the ECT (weeks), mean (±SD)	36.05 (4.32)	36.16 (3.45)	34.83 (5.51)	37.11 (4.24)	0.869	0.648
Duration of affective symptoms during the year after the ECT (weeks), mean (±SD)	21.74 (12.14)	11.58 (4.03)	18.89 (4.31)	38.14 (5.16)	23.148	<0.001 *
Li vs. Car + Val, *n* (%)	18 (100.0)	9 (50.0)	6 (33.3)	3 (16.7)	3.958	0.138
MM, *n* (%)	4 (100.0)	1 (8.3)	0 (0.0)	3 (75.0)	4.760	0.093
MDE, *n* (%)	10 (100.0)	2 (20.0)	6 (60.0)	2 (20.0)	6.500	0.039 *
FMFs/UMSs, *n* (%)	10 (100.0)	5 (50.0)	1 (10.0)	4 (40.0)	2.875	0.238
MD, *n* (%)	6 (100.0)	4 (66.7)	2 (33.3)	0 (0.0)	3.611	0.164
CS, *n* (%)	6 (100.0)	4 (66.7)	1 (16.7)	1 (16.7)	2.222	0.329

Footnote: Car—carbamazepine, CS—catatonia specifier, ECT—electroconvulsive therapy, MDE—major depressive episode, FMFs—frequent mood fluctuations, Li—lithium, MD—mixed depression, MM—mixed mania, SD—standard deviation, UMSs—unstable mood states, Val—valproate. * *p* < 0.05.

**Table 5:** Relationships between response (full and partial prophylactic effect group and no effect group) and mood stabilizing agent, gender, and syndrome (three-way ANOVA).

Factor ^a^	Df	MSq	F-Statistics	*p*-Value
Therapy (Li, Car + Val)	1	0.587	3.971	0.064
Gender (female, male)	1	0.017	0.113	0.741
Index Syndrome (MM, MDE, FMFs, MD)	3	0.586	3.962	0.027 *
Therapy–Gender	1	0.013	0.087	0.772
Therapy–Syndrome	3	0.224	1.514	0.249
Gender–Syndrome	3	0.123	0.831	0.496

Footnote: ^a^—dependent variable: response (full and partial prophylactic effect group and no effect group), ANOVA—analysis of variance, Car—carbamazepine, Df—degree of freedom, FMFs—frequent mood fluctuations, Li—lithium, MD—mixed depression, MDE—major depressive episode, MM—mixed mania, MSq—mean square, Val—valproate. * *p* < 0.05.

## Data Availability

The data of the original research described herein are available from the first author S.M. upon reasonable request.
